# Editorial: Impact of school air quality on children's health and academic performance

**DOI:** 10.3389/fpubh.2025.1707742

**Published:** 2025-10-14

**Authors:** Julia C. Fussell, Maria Salomé Giao, Ioar Rivas

**Affiliations:** ^1^Environmental Research Group, Medical Research Council (MRC) Centre for Environment & Health, School of Public Health, Imperial College London, London, United Kingdom; ^2^SharkNinja Operating LLC, London, United Kingdom; ^3^Instituto de Diagnostico Ambiental y Estudios del Agua (IDAEA), Consejo Superior de Investigaciones Científicas (CSIC), Barcelona, Spain

**Keywords:** air quality, health, learning, schools, wellbeing

## 1 Introduction

Air pollution does not affect everyone equally owing to differences in both physiological susceptibility and risk of exposure. Children are particularly susceptible to harm because of immature and developing organ systems, increased pulmonary ventilation and a compromised ability to deal with toxic compounds. Impacts of air pollution exposure on children include reduced lung function, asthma, respiratory infections, neurodevelopmental disorders, behavioral issues and increased risk of obesity. Adverse exposures during childhood and adolescence can also lead to poorer health outcomes throughout the lifecourse. Between the ages of five and 18, children spend 5–8 h a day at school. Unfortunately, in schools children can be exposed to suboptimal air quality and, consequently, experience detrimental effects on health and cognitive function. This Research Topic aimed to increase knowledge of such an impact, thereby adding to an evidence base that can be drawn upon to help adopt targeted interventions to ensure healthy environments that enable children to thrive physically and mentally.

## 2 Contributions to the Research Topic

This Research Topic contains five peer-reviewed original papers covering varying international contexts (China, Eastern and Western Europe and the USA) and approaches to assess the importance of good air quality in and around schools.

### 2.1 Long-term exposure to air pollution and lung function among children

Teng et al. investigated the relationship between lung function in 617 school children and long-term concentrations of ambient air pollutants near schools in low, moderate and high polluted areas of Anhui province, China. Forced vital capacity (FVC), forced expiratory volume in one second (FEV_1_) and forced expiratory flow between 25 and 75% (FEF_25 − 75_) showed inverse trends with increasing air pollution concentrations. The negative associations were more marked for children who were younger, female, not exposed to secondhand smoke, not overweight, physically inactive and deficit in vitamin D. Children exposed to high air pollutant concentrations and secondhand smoke had the worst lung function.

### 2.2 Upper respiratory infections in children and concentrations of vanadium in indoor dust aggregates

Prokopciuk et al. investigated which trace elements (As, Cr, Cu, Mn, Ni, Pb, Sb, Sn, V, W, Zn, Zr) within classroom dust aggregates collected from 11 primary schools in Vilnius, Lithuania were related to the annual incidence of physician-diagnosed acute upper respiratory infections among 6–11-year-old children of each school. A significant correlation was observed between the concentration of vanadium (but not other trace elements) in the dust samples collected and the incidence of acute upper respiratory infections. Higher concentrations of vanadium were present in dust from schools located in suburbs where there were industrial facilities, many homes using stove heating and/or a high density of traffic.

### 2.3 Air pollution in primary schools and children fatigue

Taminskiene et al. focused on associations between self-reported fatigue in 547 children from eight primary schools in Vilnius, Lithuania and the composition [particulate matter (PM), As, Ba, Br, Cr, Cu, Mn, Ni, Pb, Rb, Sb, Sn, Sr, V, W, Zn, Zr] of classroom dust samples. Children's fatigue was linked to worse overall health, lower academic performance, and fewer after-class activities. Significant (although relatively weak) correlations were found between higher lethargy in children and higher concentrations of PM_2.5_ and PM_1_ (PM less than 2.5 and 1 μm), copper and vanadium in the natural dust aggregates.

### 2.4 Teacher behaviors, air change rates, and use of air purifiers—influence on indoor air quality in naturally ventilated schools

Xia et al. conducted community-based participatory research in two schools in Detroit, Michigan, USA involving indoor air quality (IAQ) assessments and teacher and staff surveys before and after the installation of air purifiers with usage monitors. Baseline measurements revealed high concentrations of carbon dioxide, low air change rates and PM_2.5_ and black carbon indoor/outdoor ratios reflecting little attenuation of outdoor emissions from traffic and industry. Baseline surveys indicated a lack of awareness of IAQ concerns. Although air purifier operation substantially lowered particulate pollutants, measured removal rates varied widely among classrooms, reflecting differences in teacher behavior (e.g., frequency of window opening, purifier fan speed settings, use of purifiers at night). Increased use of purifiers appeared to correlate with teachers who were engaged in the program and more satisfied with environmental conditions.

### 2.5 Role of environmental and socioeconomic factors on children's health, learning, and inequalities—stakeholder perspectives

The study conducted by Daniels et al. in Greater Manchester, UK, found by way of semi-structured interviews that teachers and local government representatives view environmental factors (e.g., air pollution), socio-economic factors (e.g., deprivation) and poor housing as having a significant impact on children's health, development, and learning. Significant concern was voiced over the rise in Special Educational Needs and cognitive issues and how environmental factors may play a role. While local initiatives such as School Streets and active travel programs exist, their effectiveness is mixed due to multiple challenges (i.e., funding, parental resistance, lack of evaluation data).

## 3 Implications for future research and practice

Findings from this Research Topic support several common recommendations to improve IAQ in schools. These not only include more stringent and evidence-based air pollution control measures, adequate/appropriate ventilation and cleaning, regular monitoring of IAQ but also integration of air quality into school health policies, coordinated through effective engagement and collaboration among teachers and school staff, authorities, parents and caregivers. Adoption of such measures are further supported by studies reporting that policies designed to reduce exposure to air pollution can improve the health, educational performance and school attendance amongst children. It is encouraging therefore to witness the advent of initiatives such as increased air quality monitoring in schools, clean air accreditation schemes and citizen science campaigns. Papers in this Research Topic also call for deeper understanding of the link between indoor and ambient air quality in schools and its surroundings and children's health, wellbeing and performance. This in turn requires high-quality and large-scale research that can (a) inform future policy, building and street design strategies and investment decisions for schools and (b) foster long-term behavioral changes among school staff and pupils to create school environments that support children's health and educational success.

